# Several Different Lactase Persistence Associated Alleles and High Diversity of the Lactase Gene in the Admixed Brazilian Population

**DOI:** 10.1371/journal.pone.0046520

**Published:** 2012-09-28

**Authors:** Deise C. Friedrich, Sidney E. B. Santos, Ândrea K. C. Ribeiro-dos-Santos, Mara H. Hutz

**Affiliations:** 1 Departamento de Genética, Universidade Federal do Rio Grande do Sul, Porto Alegre, Brazil; 2 Laboratório de Genética Humana e Médica, Universidade Federal do Pará, Belém, Brazil; Vanderbilt University, United States of America

## Abstract

Adult-type hypolactasia is a common phenotype caused by the lactase enzyme deficiency. The −13910 C>T polymorphism, located 14 Kb upstream of the lactase gene (*LCT*) in the *MCM6* gene was associated with lactase persistence (LP) in Europeans. This polymorphism is rare in Africa but several other variants associated with lactase persistence were observed in Africans. The aims of this study were to identify polymorphisms in the *MCM6* region associated with the lactase persistence phenotype and to determine the distribution of *LCT* gene haplotypes in 981 individuals from North, Northeast and South Brazil. These polymorphisms were genotyped by PCR based methods and sequencing. The −13779*C,−13910*T, −13937*A, −14010*C, −14011*T LP alleles previously described in the *MCM6* gene region that acts as an enhancer for the *LCT* gene were identified in Brazilians. The most common LP allele was −13910*T. Its frequency was highly correlated with European ancestry in the Brazilian populations investigated. The −13910*T was higher (0.295) in southern Brazilians of European ancestry and lower (0.175) in the Northern admixed population. *LCT* haplotypes were derived from the 10 *LCT* SNPs genotyped. Overall twenty six haplotypes previously described were identified in the four Brazilian populations studied. The Multidimensional Scaling analysis showed that Belém, in the north, was closer to Amerindians. Northeastern and southern Afro-descendants were more related with Bantu-speaking South Africans whereas the Southern population with European ancestry grouped with Southern and Northern Europeans. This study shows a high variability considering the number of *LCT* haplotypes observed. Due to the highly admixed nature of the Brazilian populations, the diagnosis of hypolactasia in Brazil, based only in the investigation of the −13910*T allele is an oversimplification.

## Introduction

Adult-type hypolactasia or lactose intolerance (OMIM #223100) is a worldwide common phenotype determined by lactase deficiency, it is due to lactase activity decline after weaning. Lactase or lactase-phlorizin hydrolase enzyme (EC 3.2.1.23-62) is encoded by the *LCT* gene and it is located in the brush border membrane of small-intestinal enterocytes. The lactase enzyme activity is to hydrolyze lactose, the main carbohydrate in milk [1]. Most intolerant subjects present symptoms like bloating, flatulence, nausea, and diarrhea after consumption of fresh milk [2]–[4]. Moreover, adult-onset lactase decline appears to be a risk factor for osteoporosis due to avoidance of dairy products or undigested lactose interference with calcium absorption [5].

The regulation of *LCT* expression in humans has been studied extensively. No causative differences in the *LCT* gene sequence have been found within the gene. However, a T/C polymorphism at position −13910 and an A/G polymorphism at position −22018 from the start codon of the *LCT* gene have been identified. Although these nucleotide variants are located in introns 9 and 13 of the neighboring *MCM6* gene, the −13910*C allele associates 100% and the −22018*G allele associates approximately 97% with the lactase nonpersistent phenotype [6]–[8]. The region surrounding the −13910 position has been described to function as an enhancer stimulating the *LCT* promoter activity. The derived allele −13910T increases promoter activity [8]–[11].

The *LCT* gene (OMIM #603202) was mapped on 2q21 [12]. Several single nucleotide polymorphisms (SNPs) were described across the lactase gene, and these polymorphic sites were used to derive *LCT* haplotypes [13], [14]. The two SNPs associated with lactase persistence (LP) phenotype are linked to an A-haplotype background in European populations [6]. Mulcare et al. [15] showed that the −13910*T allele cannot be causal of lactase persistence in most Africans, although it could possibly explain lactase persistence in some Cameroonians. In that study, it was suggested that the presence of the −13910*T allele in Cameroon is due to introgression from outside sub-Saharan Africa.

In nomadic pastoralist and non-pastoralists groups from East and South Africa and Middle East populations, other polymorphisms at the same enhancer region or on its vicinity were also related to the LP phenotype. For example, −13907C>G (rs41525747) and −13915T>G (rs41380347) were both identified in Ethiopia, Kenya, Saudi Arabia, Sudan, and Tanzania populations, whereas the −13915T>G was also found in Ethiopian Somali, Morocco, and Jordan [16]–[19], whereas the −14010G>C (rs145946881) polymorphism was described in Kenya, Tanzania and Xhosa-speaking South Africans [17], [20]. Functional studies demonstrated the role of the −13910*T, −13907*G, −13915*G, and −14010*C alleles in the maintenance of the enzyme expression during adulthood [9]–[11].

The Brazilian populations were formed by successive migratory waves. Amerindian people occupied the Brazilian territory when the Portuguese arrived in 1500 and colonized the country. Then between the 16^th^ and 19^th^ centuries, West and Southwest Africans were brought to Brazil as slaves. In addition to the Portuguese, other migratory waves occurred in the 19^th^ and 20^th^ centuries, mainly from Italy, Germany and Spain [21]. All of these migratory events contributed to the formation of a multi-ethnic and highly admixed population. This heterogeneity was documented in several genetic studies that used uniparental or autosomal markers to demonstrate a typical, although non-uniform, tri-ethnic (European, African and Amerindian) pattern for the Brazilian population. This admixture process occurred in different ways in the various geographic regions of the country. In Northeastern Brazil, the African contribution is high; in the North, the contribution of Native Americans is pronounced; and in the South, there are reduced Amerindian and African influences when compared with the other geographic regions [22], [23].

The aims of this study were (1) to determine the prevalence of LP related alleles; and (2) to describe the distributions patterns of the *LCT* haplotypes in the Brazilian population.

## Results

### Identification of SNPs in the LCT Enhancer Region

The overall −13910*T allele frequency varied from 17.5% in the Northern admixed population to 29.5% in Southern Brazilians of European ancestry (Table 1). The −13910*T allele frequency is higher in the Southern Euro-descendants (p = 1.7×10^−5^) than in the other Brazilian populations investigated. As LP is a dominant trait [24], the LP predicted phenotype frequency based on −13910C>T genotypes was inferred (Table 1). The majority of the population from Belém, Recife, and the Afro-descendants from Porto Alegre are lactose intolerant (CC genotype with almost 70% frequency). In the Euro-descendants individuals from Porto Alegre, the lactase persistence frequency is higher than 50%.

**Table 1:** Observed allele and genotype frequencies of the −13910 C>T polymorphism in four Brazilian populations.

	−13910*T allele	−13910 C>T genotypes			LP predicted phenotype frequency
Population		TT	CT	CC	
		N %	N %	N %	
Porto Alegre^a^					
European ancestry (n = 337)	0.295	26 7.7	147 43.6	164 48.6	0.513
African ancestry (n = 182)	**0.184**	9 4.9	49 26.9	124 68.1	0.320
Belém^b^ (n = 200)	**0.175**	8 4.0	54 27.0	138 69.0	0.310
Recife^c^ (n = 262)	**0.204**	11 4.2	85 32.4	166 63.4	0.366

n = number of individuals; the genotypes are at Hardy-Weinberg equilibrium.

a Southern Brazil.

b Northern Brazil.

c Northeastern Brazil.

Frequencies in bold are lower than the frequency observed in individuals with European ancestry from Porto Alegre. Heterogeneity Chi-square test: p = 1.7×10^−5^).

Results from Chi-square test partition according to Everrit [50].

p = 2×10^−4^ for the comparison between individuals from European and African ancestries from Porto Alegre.

p = 2.2×10^−5^ for the comparison between population from Belém and from Porto Alegre with European ancestry.

p = 0.001 for the comparison between population from Recife and from Porto Alegre with European ancestry.

Comparisons of the −13910*T allele frequencies of the four Brazilian populations with frequencies available in dsSNP-NCBI database showed that overall these frequencies differ from those described, although some similarities could also be observed. Subjects with European ancestry from Porto Alegre are similar to those described at the global frequency from the NIH Polymorphism Discovery Resource (PDR90) only. The Afro-descendants from Porto Alegre, Recife, and Belém populations did not differ from African Americans from Southwest USA (HapMap-ASW). The Recife population also did not differ from the frequency described at the 1000Genomes project. These results are shown in the Table S1.

The −13910C>T and −22018G>A polymorphisms are in high linkage disequilibrium (data not shown). The combined frequencies of these two alleles are presented in Table 2.

**Table 2:** Frequencies of the combinations found between the −13910 C>T and −22018 G>A alleles in the Brazilian population.

	Porto Alegre		Belém	Recife
Alleles	European ancestry (n = 337)	African ancestry (n = 182)	(n = 200)	(n = 262)
CG	0.686	0.811	0.822	0.784
TA	0.279	0.184	0.175	0.204
CA	0.022	0.005	0.003	0.008
TG	0.013	–	–	0.004

Table 3 shows other variants found by sequencing of the *LCT* enhancer region in the Brazilian population. Four different polymorphisms were observed: −13937*A (rs4988234), −14010*C (rs145946881), −14011*T (rs4988233) and −13779*C (not included in the dsSNP). Their regional distribution is also shown in Table 3. A total of 9 heterozygous individuals for each of these alleles were detected. The Northeastern population was the more variable with 3 different alleles besides the −13910*T whereas Euro-descendants subjects from the South presented only the −13910*T allele.

**Table 3:** Number of −13779*C, −13937*A, −14010*C, and −14011*T alleles according to the population.

Allele	Porto Alegre African ancestry	Belém	Recife
−13779*C			2^d^
−13937*A	1^a^		1^e^
−14010*C	1^b^		
−14011*T		1^c^	3^f^

a Heterozygous A and U *LCT* haplotypes.

b Heterozygous A and P *LCT* haplotypes.

c Homozygous A *LCT* haplotype.

d Heterozygous C and U *LCT* haplotypes; heterozygous C and B *LCT* haplotypes.

e Heterozygous A and g *LCT* haplotypes.

f Homozygous A *LCT* haplotype; heterozygous A and E *LCT* haplotypes; heterozygous A and S *LCT* haplotypes.

### LCT Haplotypes

A total of 26 haplotypes were observed in the Brazilian population. The most variable population was Recife in the northeast that presented 21 haplotypes. The most frequent haplotype in all populations was the A. The haplotypes observed and their frequencies are shown in Table 4. The F_ST_ values were calculated for haplotype frequencies of the four Brazilian and their parental populations (Amerindians, Bantu-speaking population from Africa, and Southern and Northern Europeans). The degree of differentiation among populations is not high. The highest F_ST_ value is between the Euro-descendants from Porto Alegre and the Bantu-speaking population from Africa (0.202, p<0.0001, Table 5).

**Table 4:** *LCT* haplotypes frequencies ± standard error in the Brazilian population.

	Porto Alegre		Belém	Recife
*LCT* haplotype^a^	European ancestry (n^b^ = 321)	African ancestry (n = 182)	(n = 200)	(n = 258)
A	0.460±0.019	0.343±0.025	0.380±0.024	0.378±0.021
B	0.194±0.015	0.127±0.017	0.147±0.018	0.168±0.016
C	0.176±0.015	0.225±0.022	0.242±0.021	0.205±0.018
D	0.028±0.006	0.011±0.005	0.025±0.008	0.008±0.004
E	0.060±0.009	0.036±0.009	0.055±0.011	0.029±0.007
G	0.001	0.003	0.005±0.003	0.002
H			0.005±0.003	0.002
I	0.005±0.003	0.008±0.005	0.003	0.010±0.004
J	0.003±0.002	0.003		0.012±0.005
K	0.005±0.003	0.036±0.009	0.012±0.005	0.025±0.007
M	0.001	0.003	0.003	0.002
P	0.014±0.005	0.038±0.01	0.030±0.008	0.023±0.006
Q	0.005±0.003	0.008±0.005	0.003	0.012±0.005
S	0.006±0.003	0.044±0.01	0.017±0.006	0.015±0.005
T			0.003	0.002
U	0.030±0.007	0.066±0.013	0.045±0.010	0.062±0.010
W	0.001			
X	0.008±0.003	0.030±0.009	0.025±0.008	0.027±0.007
C	0.001	0.008±0.005		0.002
D	0.001			
G				0.010±0.004
H		0.003		
M				0.004±0.003
O		0.005±0.004		0.002
P	0.001			
Q		0.003		

If only one chromosome was identified, the standard error was not estimated.

a Nomenclature according to Hollox et al. [14].

b n = number of individuals.

**Table 5:** F_ST_ values of haplotype frequencies among Brazilians and with the parental populations (Amerindians, Bantu-speaking population from Africa, Southern and Northern Europeans).

	Porto Alegre		Belém	Recife
	African ancestry	European ancestry		
Porto Alegre European ancestry	**0.01981**			
Belém	0.00202	0.00796		
Recife	0.00055	**0.00861**	0.00047	
Brazilian Amerindian^a^	**0.03714**	**0.06281**	**0.02874**	**0.04412**
North Europe^b^	**0.19868**	**0.13396**	**0.18029**	**0.17467**
South Europe^b^	**0.02886**	**0.01916**	**0.02607**	0.01738
Bantu^b^	**0.10271**	**0.20200**	**0.14165**	**0.13205**

F_ST_ values in bold are statistically significant (p<0.0001).

a Haplotype frequencies from Friedrich et al. [26].

b Haplotype frequencies from Hollox et al. [14].

**Figure 1:**
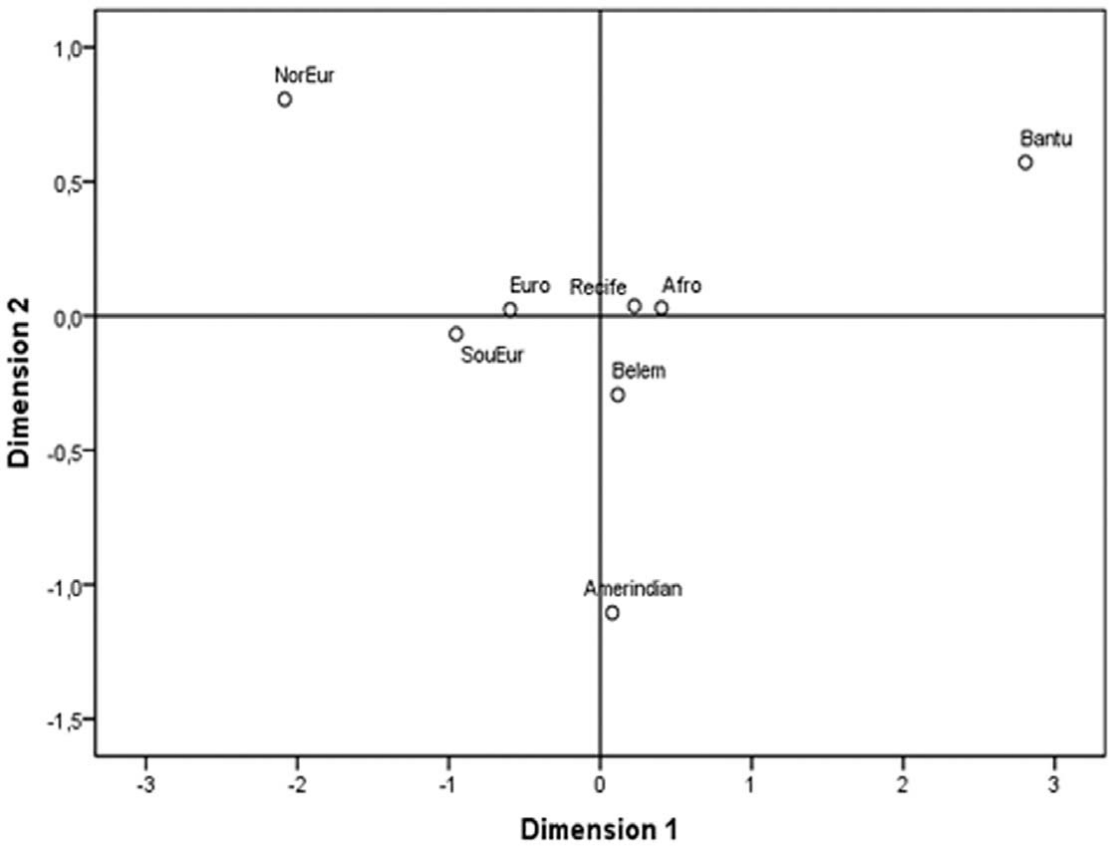
MDS of several populations *LCT* haplotypes. Nonmetric Multidimensional Scaling analysis of *LCT* haplotypes based on D_A_ distance showing the relationships among the four Brazilian populations with their parental groups: Brazilian Amerindians [26]; Southern Europeans, Northern Europeans, and Bantu-speaking South Africans [14]. NorEur  =  Northern European, SouEur  =  Southern European, Euro  =  Porto Alegre Euro-descendant, Afro  =  Porto Alegre Afro-descendant.

The nonmetric Multidimensional Scaling (MDS) analysis (Figure 1) showed the relationships among Brazilians with their parental populations based on D_A_ genetic distance. Belém, in the north, was closer to Amerindians. Recife and southern Afro-descendants were more related with Bantu-speaking South Africans whereas the Porto Alegre population with European ancestry grouped with Southern and Northern Europeans. The stress of this model is 0.047.

The −13910*T allele was observed in other haplotypes than the A in the Brazilian population. The association of the −13910*T and *LCT* haplotypes in the four Brazilian populations are shown in the Tables S2, S3, S4, and S5.

## Discussion

The −13910*T allele is present in the three Brazilian regions studied. The highest frequency of this allele was found in Euro-descendants southern subjects. The Afro-descendants from Porto Alegre also have a high frequency of this allele (18.4%) probably due to the high proportion of European contribution (43.1%) [23]. The −13910*T LP European allele was also present in Northern and Northeastern Brazil, both populations with high contributions of European ancestry to their gene pool (69.7% and 60.6%, respectively) [23].

The −13910C>T and −22018G>A polymorphisms are in high linkage disequilibrium in the four Brazilian populations studied, as in Northern Europeans and in the African Fulbe population [6], [25]. The G → A mutation might have occurred only shortly before the C → T mutation and it has been suggested that there was not enough time for recombination to break the TA haplotype [25]. Nevertheless we found the −13910*T allele in combination with the −22018*G allele in southern Brazilians with European ancestry and in the admixed population from the northeast (Tables S2, and S5). This combination would be possible under three situations: if a *de novo* mutation generating a −13910*T allele occurred in a chromosome that carried a −22018*G allele; or if a recombination event occurred; this second situation being more plausible in a highly admixed population as Brazilians. Moreover the fact that the TG combination is present in four different haplotypes (Tables S2, and S5) reinforces the recombination hypothesis. Another possibility for this finding is the contribution of Amerindian genomic ancestry. The TG haplotype was also observed in this ethnic group [26].

The CA combination was found on an A-haplotype background (Tables S2, S3, S4, S5). This CA haplotype is considered rare but it was also reported in the population from London [27], Portugal, São Tomé Island [25], and Kaingang [26]. It is interesting that the São Tomé Island has a colonization history similar to Brazil, where Portuguese settlers imported slaves from the Gulf of Guinea and from Congo and Angola region [28]. Maybe the CA combination is a genomic vestige of the Portuguese settlers that colonized both São Tomé Island and Brazil.

The −13910*T allele has been first reported to occur exclusively on a *LCT* A haplotype background [27]. More recently, it has been shown that the −13910*T occurs on two divergent A subhaplotypes suggesting more than one origin for the lactase persistence allele in Europeans [29]. Ingram et al [19] described this allele on an F haplotype in this ethnic group. In Brazil the −13910*T allele was found on A, B, J, and K haplotypes backgrounds (Tables S2, S3, S4, S5). Probably recombination was the source of this diversity in this tri-ethnic population.

This study reports the presence of other substitutions in the *LCT* enhancer region than the −13910C>T in the Brazilian population. These variants had been previously described in Africans. The −14010*C allele observed in one individual with African ancestry from Porto Alegre is common in East and South Africa. This allele is considered a LP allele because it has been demonstrated *in vitro* that it increases gene transcription [30]. It occurs in 32% and 39% of Kenyans and Tanzanians respectively [18]. It has also been reported in the Somali [19]. In Black Xhosa-speaking South Africans the −14010*C allele was observed in 13.3% of the individuals investigated [20]. This allele was also observed in low frequencies (1–6%) in Angola, Southwest Africa [31]. Two explanations for these observations in different parts of Africa were hypothesized: 1) a direct migratory link between East and Southwest Africa; 2) a first contact between East African and South pastoralists, followed by −14010*C allele transfer to Southwest pastoralists [31].

In Brazil, the −14010*C allele occurred in a heterozygous individual for A and P haplotypes. This allele was first described on an F haplotype [17] and after on a B-haplotype background [19]. In this last report the −958C>T (rs56064699) polymorphism that discriminates between B and P haplotypes was not tested; therefore the B haplotype of Ingram et al. [19] might be the same P haplotype observed herein.

Less is known about the other variants detected. The −13779*C allele was described in a lactose non-digester individual (frequency 1/107) from a Somali cohort [19]. But it was common in an Indian herder sample (0.024) [32]. Now this allele was detected in two admixed subjects from Recife. The −13937*A allele was observed in one individual of African ancestry from Porto Alegre and in one individual from Recife. This allele was described at low frequencies (0.014) in the Black Xhosa-speaking people from South Africa, a population that has the habit of consuming fermented milk [20]. Functional studies about the role of these two variants in the transcription of the *LCT* have not been performed yet.

The −14011C>T was described in the Estonian and Indian population [32], [33] and its global frequency is 0.006. The functional role of this variant in the *LCT* transcription is unknown. But its location is a good predictor of functionality since it is close to the −14010G>C that interacts with transcription factors that increase lactase promoter activity [30]. This variant was observed in four admixed Brazilian subjects, three from the Northeast and one from the North.

This diversity at the *LCT* enhancer region is not unexpected if we consider the Brazilian roots. The slaves brought to Brazil were mainly from West and Southwest African areas and highly admixed with the European colonizers [23], [34].

Although the high admixture rate in Brazil determine a high number of *LCT* haplotypes, the A haplotype was the most frequent. The A haplotype prevalence is explained by the large contribution of European ancestry to the Brazilian population gene pool: 69.7% in the North, 60.6% in the Northeast, and 94% in the South [23], [34].

The MDS analysis shows the close relation of parental populations to Brazilians. Southern Europeans are closer to Porto Alegre Euro-descendants. The Porto Alegre Afro-descendants and Recife population are more related to the Bantu-speaking South Africans, whereas Brazilian Amerindians have a closer relation with the Belém population.

Two previous studies validated the screening of the −13910C>T polymorphism for hypolactasia molecular diagnosis in Brazil [35], [36]. A third study concluded that the −22018G>A polymorphism is a better predictor of lactase persistence in Japanese-Brazilians than the −13910C>T [37]. In our study we demonstrated that a more comprehensive screening would be needed since we found four variants in the enhancer region besides the −13910C>T. If only the −13910C>T polymorphism would be tested in Recife, for example, 6 individuals would be considered lactose intolerant and they are carrier of *LCT* enhancer region variants that could be causal of the lactase persistence phenotype. In heterogeneous populations like Brazilians a single test for the −13910C>T polymorphism is an underestimate of the LP phenotype.

**Figure 2:**
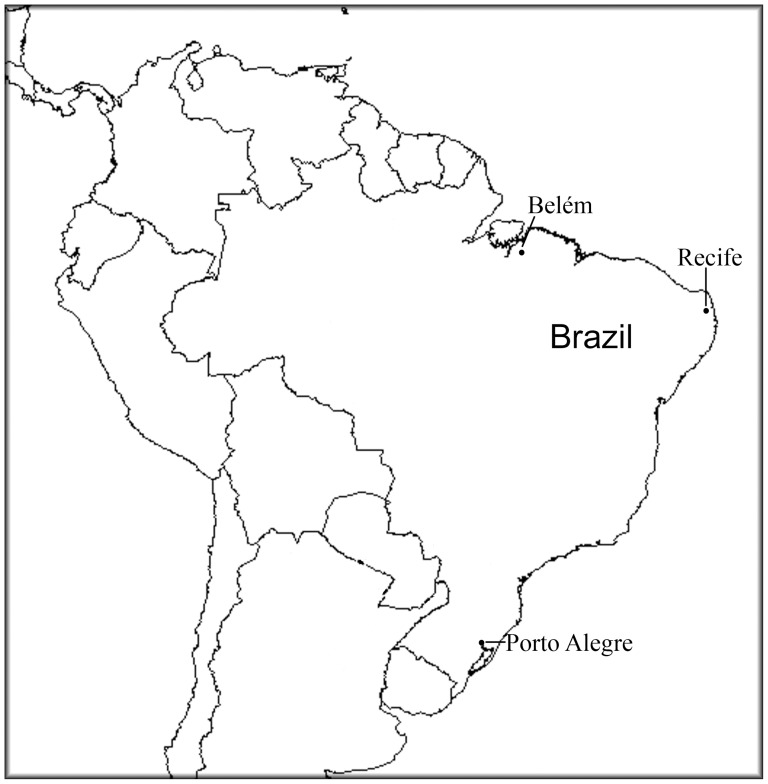
Geographic location of the 3 Brazilian cities where the samples were collected.

## Materials and Methods

### Ethics Statement

All enrolled subjects provided their written informed consent to participate. The study protocol was approved by the ethics committees of the Federal University of Rio Grande do Sul, Federal University of Pará and of the Instituto Materno Infantil de Pernambuco, the three institutions that participated in blood sample collection.

### Subjects

The study cohort consisted of 981 individuals recruited from the North, Northeast, and South regions of Brazil (Figure 2). All individuals from the Southern sample were selected at random at the Clinical Analysis Laboratory of the Pharmacy School of the Federal University of Rio Grande do Sul, Porto Alegre, among those who came from several city health centers for free routine blood determinations. This sample included 337 individuals of European ancestry and 182 African Brazilians. European and African ancestry were ascertained by visual inspection of skin color and morphological characteristics. These samples have been fully described in previous publications [38]–[40]. The Northeastern sample consisted of 262 healthy adolescents ascertained at Instituto Materno-Infantil de Pernambuco at Recife, the capital of the Brazilian Northeastern state of Pernambuco. The characteristics of this sample were previously described [41]. The Northern sample consisted of 200 individuals from the city of Belém in the Brazilian Amazon region ascertained at the Federal University of Pará. We did not stratify by ethnicity the Northeastern and Northern populations because skin color is not very indicative of genomic ancestry in these populations [23], [34]. The LP status of the individuals sampled was not evaluated.

### Identification of SNPs in the LCT Enhancer Region

The −13910C>T (rs4988235) polymorphism was genotyped by PCR-RFLP as previously described [35]. In an attempt to identify other polymorphisms in the −14 kb region, a fragment of 427 bp was amplified with the MCM6i13 and LAC-CL2 primers [16] in all non-carriers of the −13910*T allele. PCR products were purified with *Exonuclease* I and Shrimp Alkaline Phosphatase enzymes and then sequenced at MACROGEN (Seoul, Republic of Korea) using the MCM6i13 primer. The −22018G>A (rs182549) polymorphism was genotyped by allelic discrimination using TaqMan assays in a real time PCR equipment (StepOne Plus, Applied Biosystems).

### LCT Haplotypes

From the eleven polymorphisms described across the 70 Kb *LCT* gene [42], ten were genotyped in the present study to infer *LCT* haplotypes. Seven out of 10 polymorphisms investigated mapped between −1099 and −502 at the 5′ flanking region and were identified by sequencing a fragment of 597 pb at MACROGEN as previously described [26]. The other three polymorphisms 666G>A (rs3754689), 5579T>C (rs2278544), and 6236TG>ΔΔ (rs10552864) reside within the *LCT* gene, and were genotyped by TaqMan assays. The haplotypes were designated according to the nomenclature previously described [14].

### Statistical Analysis

Chromatograms were examined using CodonCode Aligner Software v.3.5.7. Allele frequencies were obtained directly by gene counting. Hardy-Weinberg equilibrium was tested by Chi-Square with the WINPEPI software [43]. This software was also used for allele frequency comparisons by heterogeneity Chi-Square test. *LCT* haplotypes were inferred using a Bayesian algorithm implemented in Phase v.2.1 software [44], [45]. Wrigh’s F_ST_ [46], [47] was calculated using Arlequin v.3.0 [48]. D_A_ distance [49] matrix for the *LCT* haplotypes frequencies was generated with POPTREE software version 1. This matrix was used in the nonmetric Multidimensional Scaling analysis performed with SPSS v.18.

## Supporting Information

Table S1 P-values from the Chi-square test of the −13910*T allele frequencies among the studied populations from Brazil and the data available in dsSNP-NCBI.
(DOC)

Table S2 **Distribution of the −13910 C>T and −22018 G>A haplotype on the** ***LCT*** **haplotypes in the Euro-descendants from Porto Alegre city, Rio Grande do Sul State, Brazil.**
(DOC)

Table S3 **Distribution of the −13910 C>T and −22018 G>A haplotype on the** ***LCT*** **haplotypes in the Afro-descendants from Porto Alegre city, Rio Grande do Sul State, Brazil.**
(DOC)

Table S4 **Distribution of the −13910 C>T and −22018 G>A haplotype on the** ***LCT*** **haplotypes in the Belém city population, Pará State, Brazil.**
(DOC)

Table S5 **Distribution of the −13910 C>T and −22018 G>A haplotype on the** ***LCT*** **haplotypes in the Recife city population, Pernambuco State, Brazil.**
(DOC)
