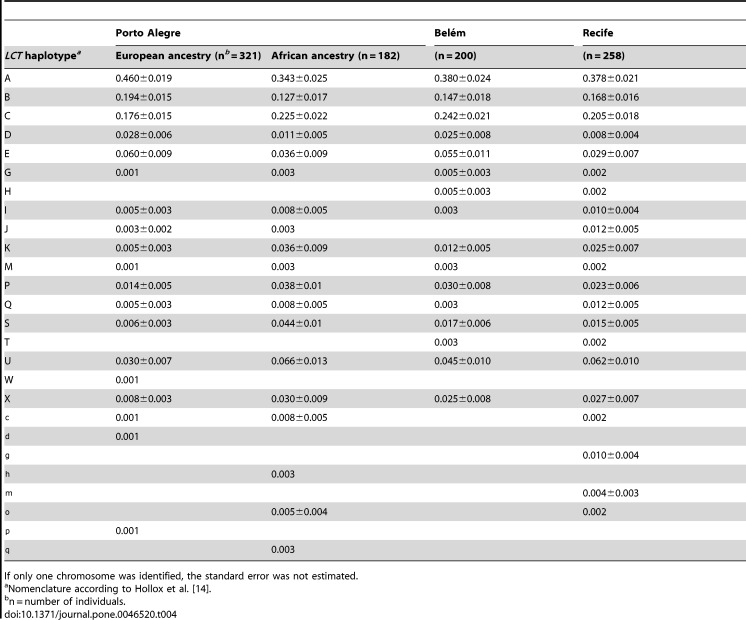# Correction: Several Different Lactase Persistence Associated Alleles and High Diversity of the Lactase Gene in the Admixed Brazilian Population

**DOI:** 10.1371/annotation/04ccbcf8-8910-43b6-8d25-b2ca0270cd84

**Published:** 2013-10-16

**Authors:** Deise C. Friedrich, Sidney E. B. Santos, Ândrea K. C. Ribeiro-dos-Santos, Mara H. Hutz

In Table 4, the letters c-q near the bottom of "LCT haplotype" were uppercase when they should be lowercase. Please see the corrected Table 4 here: 

**Figure:**