# Supplementary material for: Several Different Lactase Persistence Associated Alleles and High Diversity of the Lactase Gene in the Admixed Brazilian Population
Source: PLoS One. 2012 Sep 28;7(9):e46520. doi: 10.1371/journal.pone.0046520 (PMC3460917; doi:10.1371/journal.pone.0046520)
Supplement: Table S1 — P-values from the Chi-square test of the −13910*T allele frequencies among the studied populations from Brazil and the data available in dsSNP-NCBI. (DOC) [file pone.0046520.s001.doc]

Table S1. P-values from the Chi-square test of the -13910*T allele frequencies among the studied populations from Brazil and the data available in dsSNP-NCBI.

|  | Porto Alegre | | Belém | Recife |
| --- | --- | --- | --- | --- |
|  | European ancestry | African ancestry |  |  |
| HapMap-CEU*^a^* | 1x10^-8^ | 9x10^-9^ | 9.3x10^-9^ | 9.7x10^-9^ |
| HapMap-ASW*^b^* | 2.5x10^-5^ | 0.277 | 0.399 | 0.079 |
| 1000Genomes*^c^* | 0.001 | 0.038 | 0.01 | 0.157 |
| PDR90*^d^* | 0.712 | 0.015 | 5.6x10^-3^ | 0.048 |

*^a^* Utah residents with Northern and Western European ancestry from the CEPH collection; -13910*T allele frequency: 0.73; number of samples: 180

*^b^* Individuals with African ancestry in Southwest USA; -13910*T allele frequency: 0.143; number of samples: 90

*^c^* 1000Genome phase 1 genotype data, released in the May 2011 dataset; -13910*T allele frequency: 0.233; number of samples: 1094

*^d^* The NIH Polymorphism Discovery Resource; -13910*T allele frequency: 0.276; number of samples: 90
