# Supplementary material for: Several Different Lactase Persistence Associated Alleles and High Diversity of the Lactase Gene in the Admixed Brazilian Population
Source: PLoS One. 2012 Sep 28;7(9):e46520. doi: 10.1371/journal.pone.0046520 (PMC3460917; doi:10.1371/journal.pone.0046520)
Supplement: Table S2 — Distribution of the −13910 C>T and −22018 G>A haplotype on the LCT haplotypes in the Euro-descendants from Porto Alegre city, Rio Grande do Sul State, Brazil. (DOC) [file pone.0046520.s002.doc]

Table S2. Distribution of the -13910 C>T and -22018 G>A haplotype on the *LCT* haplotypes in the Euro-descendants from Porto Alegre city, Rio Grande do Sul State, Brazil.

| *LCT* haplotype | CG | TA | CA | TG |
| --- | --- | --- | --- | --- |
| A | 0.157 | 0.276 | 0.022 | 0.005 |
| B | 0.187 |  |  | 0.007 |
| C | 0.176 |  |  |  |
| D | 0.028 |  |  |  |
| E | 0.060 |  |  |  |
| G | 0.001 |  |  |  |
| I | 0.005 |  |  |  |
| J |  | 0.003 |  |  |
| K | 0.003 |  |  | 0.001 |
| M | 0.001 |  |  |  |
| P | 0.014 |  |  |  |
| Q | 0.005 |  |  |  |
| S | 0.007 |  |  |  |
| U | 0.030 |  |  |  |
| W | 0.001 |  |  |  |
| X | 0.008 |  |  |  |
| c | 0.001 |  |  |  |
| d | 0.001 |  |  |  |
| p | 0.001 |  |  |  |

Total number of individuals = 321
