# Supplementary material for: Several Different Lactase Persistence Associated Alleles and High Diversity of the Lactase Gene in the Admixed Brazilian Population
Source: PLoS One. 2012 Sep 28;7(9):e46520. doi: 10.1371/journal.pone.0046520 (PMC3460917; doi:10.1371/journal.pone.0046520)
Supplement: Table S3 — Distribution of the −13910 C>T and −22018 G>A haplotype on the LCT haplotypes in the Afro-descendants from Porto Alegre city, Rio Grande do Sul State, Brazil. (DOC) [file pone.0046520.s003.doc]

Table S3. Distribution of the -13910 C>T and -22018 G>A haplotype on the *LCT* haplotypes in the Afro-descendants from Porto Alegre city, Rio Grande do Sul State, Brazil.

| *LCT* haplotype | CG | TA | CA |
| --- | --- | --- | --- |
| A | 0.157 | 0.181 | 0.005 |
| B | 0.127 |  |  |
| C | 0.225 |  |  |
| D | 0.011 |  |  |
| E | 0.036 |  |  |
| G | 0.003 |  |  |
| I | 0.008 |  |  |
| J |  | 0.003 |  |
| K | 0.036 |  |  |
| M | 0.003 |  |  |
| P | 0.038 |  |  |
| Q | 0.008 |  |  |
| S | 0.044 |  |  |
| U | 0.066 |  |  |
| X | 0.030 |  |  |
| c | 0.008 |  |  |
| h | 0.003 |  |  |
| o | 0.005 |  |  |
| q | 0.003 |  |  |

Total number of individuals = 182
